# Providing surgery in a war-torn context: the Médecins Sans Frontières experience in Syria

**DOI:** 10.1186/s13031-015-0064-3

**Published:** 2015-12-15

**Authors:** Miguel Trelles, Lynette Dominguez, Katie Tayler-Smith, Katrin Kisswani, Alberto Zerboni, Thierry Vandenborre, Silvia Dallatomasina, Alaa Rahmoun, Marie-Christine Ferir

**Affiliations:** Médecins Sans Frontières-Operational Centre Brussels (MSF-OCB), Surgery, Anaesthesia, Gynaecology, and Emergency Medicine Unit, Brussels, Belgium; MSF-OCB, Operational Research Unit, MSF-Luxembourg, Luxembourg, Luxembourg; MSF-OCB, Emergency pool, Brussels, Belgium; MSF-OCB, Logistics department, Brussels, Belgium; MSF-OCB, MSF Syria project, Latakia Governorate, Syria

**Keywords:** Quality surgical care, Conflict settings, Syria, Minimum standards of care

## Abstract

**Background:**

Since 2011, civil war has crippled Syria leaving much of the population without access to healthcare. Various field hospitals have been clandestinely set up to provide basic healthcare but few have been able to provide quality surgical care. In 2012, Medecins Sans Frontieres (MSF) began providing surgical care in the Jabal al-Akrad region of north-western Syria. Based on the MSF experience, we describe, for the period 5th September 2012 to 1st January 2014: a) the volume and profile of surgical cases, b) the volume and type of anaesthetic and surgical procedures performed, and c) the intraoperative mortality rate.

**Methods:**

A descriptive study using routinely collected MSF programme data. Quality surgical care was assured through strict adherence to the following minimum standards: adequate infrastructure, adequate water and sanitation provisions, availability of all essential disposables, drugs and equipment, strict adherence to hygiene requirements and universal precautions, mandatory use of sterile equipment for surgical and anaesthesia procedures, capability for blood transfusion and adequate human resources.

**Results:**

During the study period, MSF operated on 578 new patients, of whom 57 % were male and median age was 25 years (Interquartile range: 21–32 years). Violent trauma was the most common surgical indication (*n*-254, 44 %), followed by obstetric emergencies (*n*-191, 33 %) and accidental trauma (*n*-59, 10 %). In total, 712 anaesthetic procedures were performed. General anaesthesia without intubation was the most common type of anaesthesia (47 % of all anaesthetics) followed by spinal anaesthesia (25 %). A total of 831 surgical procedures were performed, just over half being minor/wound care procedures and nearly one fifth, caesarean sections. There were four intra-operative deaths, giving an intra-operative mortality rate of 0.7 %.

**Conclusions:**

Surgical needs in a conflict-afflicted setting like Syria are high and include both combat and non-combat indications, particularly obstetric emergencies. Provision of quality surgical care in a complex and volatile setting like this is possible providing appropriate measures, supported by highly experienced staff, can be implemented that allow a specific set of minimum standards of care to be adhered to. This is particularly important when patient outcomes - as a reflection of quality of care - are difficult to assess.

## Background

Surgical care is an integral part of any healthcare system and yet in low and middle income countries (LMICs) access to such care is often limited [1, 2]. When such contexts are in turn afflicted by conflict, the situation becomes even more dire: not only is access further hampered, but this at a time when the surgical needs of the population are likely to be greater (war-related injuries in addition to non-war related conditions such as obstetric emergencies and infections) [3].

Since 2011, Syria has been crippled by a brutal civil war resulting in what has been described by the United Nations (UN) as the worst humanitarian crisis in recent times. Approximately 220,000 people (mostly civilians) are estimated to have died since the conflict began and it is estimated that 12.2 million people are in need of humanitarian assistance, 7.6 million of whom are internally displaced [4]. Intense fighting and complicated relief operations due to political barriers have left millions of Syrians without access to essential services [5], access to health services being the greatest challenge of all [6]. Access to basic health care, including surgical care, is severely hampered by restricted ability to move, the destruction and breakdown of health infrastructure, shortage of medical supplies, lack of human resources and threats of kidnappings and killings by different armed groups on medical staff [7, 8]. Reports estimate that more Syrians have died due to health complications resulting from inadequate health care services than as a direct consequence of the conflict [4].

Various field hospitals have been clandestinely set up to provide some level of healthcare [9, 10] but very few have managed to establish the capacity to provide quality surgical care.

Médecins Sans Frontières (MSF), an international, independent, humanitarian medical organisation, provides medical assistance to vulnerable populations, including those affected by conflicts. Surgical care is an integral part of its work. Shortly after the conflict in Syria escalated into a full-scale civil war in 2012, MSF-OCB (Operational Centre Brussels) intervened in the Jabal al-Akrad region of north-western Syria, where it established a field hospital that included a surgical centre. Despite major challenges, MSF-OCB managed to provide surgery for nearly 600 patients.

To date, there have been few published studies reporting on the provision of surgical activities in conflict settings. Those that have, have generally reported on the typology of the surgical care provided [3, 11], remote models of care [12], the feasibility of performing specific surgical procedures [13, 14], and the surgical management and related outcomes for specific conditions [15, 16]. One study from Syria [17] described how a field hospital, which included surgical activities, was set up and organised, but the study only reported on 28 surgical patients. The ‘how’ of providing quality surgical care for larger numbers of patients presenting with different surgical indications in a complex war-torn context like Syria has not been formally reported on.

Based on the MSF-OCB experience of providing surgical care in the Jabal al-Akrad region of Syria between September 2012 and January 2014, this study thus aims to report on how quality surgical care can be provided in a complex conflict setting and what volume and type of surgical activities are feasible. Specific objectives were to report on a) the volume and profile of surgical cases seen, b) the volume and type of anaesthetic and surgical procedures performed and c) the intraoperative mortality rate.

## Methods

### Study design

This was a descriptive study using routinely collected MSF programme data.

### Study setting

Jabal al-Akrad is a rural mountainous region in north-west Syria close to the Turkish border, with a population of about 150,000. As a result of the civil war, the region has seen extensive fighting between armed groups and the Syrian army and also between different armed groups.

The MSF field hospital was set up and functional by the end of August 2012. Initially the hospital was organised within a cave, but in November 2012, due to insecurity (the position of the cave was considered to be too close to a frontline and military position) and limited space, the field hospital was moved and re-established in a permanent brick structure (an abandoned chicken farm) located 6 km from the Turkish border. The provision of surgical care was shifted from the cave to the chicken farm in just one day. Several days prior to this shift, a new surgical tent was erected in the chicken farm and a proportion of the emergency/ surgical care supplies and pharmacy stock was transferred to the chicken farm. On the day of the move, the operating table and operating lamp were shifted from the cave to the chicken farm together with the anaesthetist (in the first convoy) and the surgeon and emergency physician (in the second convoy); there was only a 90 min window during which surgical activities had to be put on hold. The remaining supplies at the cave were transferred to the chicken cave over the following two days.

#### MSF surgical activities

MSF adapted a standardised design for organising its surgical complex and implemented standardised procedures for surgical activities and training based on internal policy guidelines. The surgical centre comprised of one operating room (OR) in the form of an inflatable tent, together with all the necessary supportive units including an emergency department (with a resuscitation, treatment and observation area), a pre- and post-operative ward, a sterilisation unit, a basic laboratory and blood transfusion capacity.

Surgical activities were overseen by a team of experienced international staff (one surgeon, one anaesthetist, one emergency doctor and one OR nurse) and supported by a number of local nurses (recruited from the local community and trained accordingly to assist with surgical activities). Staff turnover among local personnel was minimal while international staff were rotated on a monthly basis - the whole team being replaced by a new one. On several occasions, international staff members, including those in the surgical team, had to be evacuated because of security constraints. On three occasions, the evacuation period was 15 days or longer and during these evacuations periods surgical activities were temporarily suspended. During these times, the hospital remained open and patient care and management was overseen by local health personnel.

An important part of the MSF intervention included setting up an effective referral system with appropriate services in Turkey. If a patient arrived at the field hospital in a critical condition that could not be surgically managed because of resource constraints, damage control surgery [18] was performed and the patient was referred to Turkey. Patients with head trauma were also referred. Furthermore, by providing orthopaedic surgical materials (mainly external fixators) to a nearby Syrian hospital with the human resource capacity to provide orthopaedic surgery, MSF was able to refer some of its orthopaedic cases to this hospital.

Emergency care, including surgery, was available 24 h a day every day, and all care and treatment was provided free of charge. Appropriate triage systems were put in place to try to ensure a more efficient use of limited resources and to better manage heavy caseloads during busy times. During times of ‘normal’ care, the South African Triage scale was used in the emergency department and the process of triage was task-shifted to nursing staff so that the few available doctors could concentrate their efforts on treating patients once triaged; during times of a mass casualty incident (MCI), START (simple triage and rapid treatment) [19] was implemented - a triage method used to quickly classify victims during a MCI based on the severity of their injury.

#### Minimum standards of surgical care

Table 1 summarises the pre-requisites considered by MSF to be essential for performing quality surgical interventions in Jabal al-Akrad and the measures taken to meet these conditions. There were seven pre-requisites: i) adequate infrastructure, (including protection from the external environment and appropriate electricity and lighting), ii) adequate water and sanitation provisions, (waste management being a key priority), iii) availability of all essential disposables, drugs and equipment, iv) strict adherence to hygiene requirements and universal precautions, v) mandatory use of sterile equipment for surgical and anaesthesia procedures, vi) capability for blood transfusion, and vii) adequate human resources in quantity and quality. In order to meet these minimum standards in the context of Syria, various measures had to be taken. Some of the key measures included: i) adapting the standard field hospital design - this to meet the functional/medical needs of the hospital, ensure the ‘safe’ circuit of patients in terms of hygiene standards and accommodate for the challenges around lack of space, lack of infrastructure and lack of security; ii) ensuring that highly experienced personnel were in place to oversee the design and timely implementation of these technical adaptations (medical staff - who could clearly identify the needs of the hospital - and logistics staff with extensive experience in emergency hospital design and set-up) and iii) establishing a clandestine supply line from Turkey to ensure a regular supply of logistical materials, fuel and medical supplies.

**Table 1:** Measures taken by MSF to ensure the provision of quality surgical care in Jabal Akkrad, Syria

Essential conditions for the provision of quality surgical care	Measures taken to achieve these conditions in the cave and chicken farm
Adequate infrastructure, including protection from the external environment and appropriate electricity and lighting.	• The standard design of the field hospital was adapted to meet the functional/medical needs of the hospital, to ensure the ‘safe’ circuit of surgical patients (in terms of hygiene standards) and to accommodate for the challenges around lack of quality space, lack of infrastructure and lack of security, all the while ensuring that quality care of care would not be compromised. Essential for the design and timely implementation of these technical solutions was the presence of highly experienced personnel (medical staff who could clearly identify the functional needs of the hospital and logistics staff with extensive experience in emergency hospital design and set-up). • An inflatable tent served as the operating theatre in order to ensure a minimum level of cleanliness. To ensure protection from environmental factors (such as rain, wind, dust and falling debris from the roof of the cave), plastic sheets formed a cover for the floor of the tent and wooden frames lined with plastic sheets formed the roof of the tent. The same features were put in place once the field hospital relocated to an abandoned chicken farm. • An entirely new electrical wiring system was installed, respecting all necessary electrical safety measures. In addition, two generators were installed (one as back up), to ensure a reliable electricity supply during power cuts. • Establishing the field hospital in the abandoned chicken farm in particular, required a huge coordinated global effort between the field team in Syria, headquarter teams in Brussels and a team based in Turkey: the field team selected the site (the chicken farm) and then sent photos to the emergency team in Brussels who, together with the logistics team, provided the team based in Turkey with a list of all the required materials. The Turkey based team obtained these materials through local procurement and then made the necessary arrangements to transport these materials across the Turkish border into Syria. While these preparations went on, logistics staff in the field set about designing the new hospital. This coordinated effort enabled the hospital to be set up within 3–4 days of relocating from the cave. • A clandestine supply line from Turkey was essential for obtaining the logistical equipment and materials needed to set up the field hospital
Adequate water and sanitation provisions, waste management being a key priority	• Minimum water requirements were pre-set at 100 L water/anaesthesia and 40–60 L water/person/day in the IPD. At the cave, three water points were installed: one for emergency care, one for scrubbing, and one for sterilisation; at the abandoned chicken farm, a 30,000 L water tank was already in place. To avoid potential contamination of water with previously used toxic chemicals at the farm, the tank was emptied, cleaned, disinfected with chlorine and filled again. • A new canal system was installed in just four days and water points were installed in seven areas of care-triage, consultation & emergency care, resuscitation, the inpatient department, scrubbing, sterilization, and laboratory. • Several easy-to-clean tiled toilet units with hand washing points were installed and connected up to a large soak-away pit built outside (not accessible to patients or visitors). • Surgical activities produce large quantities of organic, liquid and dangerous waste and there should be proper segregation of the waste material produced. While operating from the cave, there was only space enough for one waste pit which therefore had to serve as a refuse for all of the different types of waste. Once the hospital relocated to the abandoned chicken farm, a waste-management zone with an appropriate number of pits was built and waste could be segregated according to protocol.
Availability of all essential disposables, drugs and equipment	• A regular supply of drugs, material and equipment is an essential MSF pre-requisite in order to guarantee no ruptures in care. A clandestine supply line from Turkey ensured this, with supplies being transported across the Turkish border either by truck (with support of the Turkish Red Crescent) or hand-carried.
Strict adherence to hygiene requirements and universal precautions	• Hydro-alcoholic solution was made readily available. • A clean sufficient water supply and sanitation provisions were ensured (as outlined above). • In the absence of water hot enough to adequately clean dirty laundry, chlorine was added to the cold water supplying the washing machine. • Strict adherence to infection control protocols
Mandatory use of sterile equipment for surgical and anaesthesia procedures	• In the cave, dry-heat sterilization was installed • At the abandoned chicken farm, a gas barrel was installed in a separate and protected ventilated place, and linked to the heater of the autoclave inside the building. To generate the correct circuit of activities (from dirty, to clean, and finally to sterile) the sterilization service was provided in a large space, with two wicket gates for the “entry” and “exit” of material.
Blood transfusion capability	• In the cave, there was no blood bank in place. Even so, several blood transfusions were performed when indicated if suitable donors could be identified on the spot. • Once the surgical center moved to the abandoned chicken farm, a blood bank was set up with transfusion therapy restricted to life saving indications.
Adequate human resources in quantity and quality	• Human resource requirements focused on sufficiency in numbers and sufficiency in skill level. • Logisticians had to be recruited to organise the surgical centre, while surgical and aesthesia practitioners were needed to perform the required surgical procedures. • A recruitment call was made for expatriate staff with previous experience of working in war torn settings. The technical referents devised a simplified design for the surgical centre and then up to 70 Syrian staff (plumbers, carpenters, bricklayers etc.) were employed to rehabilitate the abandoned chicken farm into a hospital with an operating theatre (taking just four days).

### Study population

The study population included all new patients who underwent any surgical intervention at the MSF field hospital in Jabal al-Akrad between 5th September 2012 and 1st January 2014.

### Data collection and analysis

For this analysis, a surgical intervention was defined as any intervention performed in the OR that required local, regional and / or general anaesthesia. One surgical intervention corresponded with one patient entrance into the OR. The data collection tool in place was designed such that a maximum of three surgical procedures could be recorded per surgical intervention. Data on all surgical interventions were entered into a standardised logbook and transcribed into an electronic database (Microsoft Excel). These data were then aggregated at MSF-OCB headquarters and reviewed for completeness and accuracy. Data pertaining to this study were sourced from the electronic database and included the following variables: date of surgical intervention, patient gender, patient age, the American Society of Anaesthesiologists (ASA) score (a classification system for describing a patient’s pre-operative physical status, see Table 2), indication for surgery (grouped as accidental trauma, violent trauma, obstetric care and other), type of anaesthesia used (general anaesthesia without intubation, general anaesthesia with intubation, spinal anaesthesia, local/ regional anaesthesia, or combined anaesthesia), type of surgical procedure (minor, wound care, caesarean section, visceral surgery, orthopaedic surgery, other) and intra-operative outcome (dead/alive).

**Table 2:** American Society of Anaesthesiologists (ASA) score

Class 1:	→ Patient in apparent good health notwithstanding his surgical problem
	→ Limitations on activity: none
	→ Limitations on activity: none
	→ Excluded: persons at extremes of age (very young, very old)
Class 2:	→ Patient with mild systemic disease (e.g., mild hypertension, mild-moderate anaemia)
	→ Patient’s health: disease of one body system
	→ Status of underlying disease: well controlled
	→ Limitations on activity: none
	→ Danger of death: none
Class 3:	→ Patient with systemic disease severe enough to limit activity but not incapacitating
	→ Patient’s health: disease of more than one body system or one major system
	→ Limitations on activity: present but not incapacitated
	→ Danger of death: no immediate danger
Class 4:	→ Patient with severe incapacitating disease that is a constant threat to life
	→ Patient’s health: poor, with at least one severe disease
	→ Status of underlying disease: poorly controlled or end-stage
	→ Limitations on activity: incapacitated
	→ Danger of death: possible
Class 5:	→ Moribund patient not expected to survive 24 h with or without surgery
	→ Patient’s health: very poor, moribund
	→ Limitations on activity: incapacitated
	→ Danger of death: imminent
Class 6:	→ A declared brain-dead patient whose organs are being removed for donor purposes

Intraoperative mortality was defined as any death occurring during the induction of anaesthesia, the intervention itself, or the immediate recovery period (i.e., the time during which a patient is monitored and managed in a post-anaesthetic recovery unit [20]).

Data were analysed in STATA/IC version 11.0 (Stata corporation, Texas 77845, USA) using summary statistics.

### Ethics approval

The study met the Medecins Sans Frontieres Ethics review Board (Geneva, Switzerland) approved criteria for studies of routinely collected data. The study was conducted as a retrospective analysis of routine programme data, and thus informed consent was not sought from study subjects. No patient Identifying information was included in the final analysis or reported on.

## Results

### Volume and profile of surgical cases

Between 5th September 2012 and 1st January 2014, MSF performed surgery on 578 new patients. Table 3 shows the profile of these patients. Males comprised 57 % of the cases and the median overall age was 25 years (Interquartile range: 21–32 years). The majority of patients had an ASA score of 1 indicating good pre-operative physical status. Violent trauma was the most common surgical indication (*n*-254, 44 %); the remaining indications for surgery were non-violent in type, most commonly being obstetric emergencies (*n*-191, 33 %) and accidental trauma (*n*-59, 10 %).

**Table 3:** Profile of all new surgical patients managed at the MSF field hospital in Jabal al-Akrad, Syria (5th September 2012-1st January 2014)

Variable	*n* (%)
Total	578
Sex	
Female	250 (43)
Male	328 (57)
Age (years)	
0–14	50 (9)
15–29	332 (57)
30–44	145 (25)
≥45	51 (9)
Median [IQR]	25 (21–32)
ASA score	
1	483 (84)
2	69 (12)
3	19 (3)
4	6 (1)
5	1 (0.2)
Surgical indication	
Violent trauma	254 (44)
Obstetric emergencies	191 (33)
Accidental trauma	59 (10)
Non-traumatic and non-obstetric pathologies	74 (13)

*IQR* Interquartile range, *ASA* American Society of Anaesthesiologists

### Anaesthetic and surgical procedures performed

Table 4 shows the type of anaesthetic and surgical procedures performed. In total, 712 anaesthetic procedures were performed on the 578 patients (one anaesthetic per patient entrance into the OR: 578 new patient entrances and 134 re-entrances for surgical re-interventions). General anaesthesia without intubation was the most common type of anaesthesia (used in 47 % of surgical interventions), followed by spinal anaesthesia (25 %). Spinal procedures were used for 84 % of Caesarean sections (data not shown in the Table).

**Table 4:** Type of anaesthesia and surgical procedures performed at the MSF field hospital in Jabal al-Akrad, Syria (5th September 2012-1st January 2014)

Variable	*n* (%)
Type of anaesthetic procedures (*n*-712)	
General without intubation	337 (47)
Spinal	179 (25)
General with intubation	122 (17)
Local/regional	53 (7)
Combined	21 (3)
Type of surgical procedures (*n*-831)	
Minor/ Wound care	450 (54)
Caesarean-sections	153 (18)
Visceral surgery	114 (14)
Orthopaedic/specialised surgery	64 (8)
Gynaeco-obstetric surgery^a^	50 (6)

^a^Excluding caesarean sections

A total of 831 surgical procedures were performed on the 578 patients. Just over half of these were minor-wound procedures and nearly one fifth, caesarean sections.

### Intra-operative mortality

There were four intra-operative deaths among the 578 patients, equating to an intra-operative mortality rate of 0.7 %. The four deaths were among cases injured by bomb blasts: one with multiple severe traumas, one with a severe hip fracture, one with a lung laceration and haemo-peritoneum, and one with inferior vena cava and pancreatic tears. Of the 154 newborns delivered by Caesarean section, 6 (4 %) died.

## Discussion

In a conflict setting with extreme security constraints, where access to surgical care is severely restricted, MSF was able to adapt its modus-operandi and implement a model of emergency surgical care with good outcomes. Quality care was assured through strict adherence to a specific set of minimum standards of care and, over a 16 month period, MSF operated on nearly 600 patients - mainly young adults comprising slightly more males than females. Violent trauma was the most common reason for surgery, followed by obstetric emergencies which accounted for one third of all cases. Minor/wound care surgery made up just over half of all the surgical procedures performed, and Caesarean sections one fifth. The intra-operative mortality rate was low at 0.7 %.

The strengths of this study were that it included i) a large number of patients, ii) routine collection of data in the field hospital was thorough and standardised, and iii) the study adhered to the STROBE (Strengthening the Reporting of Observational Studies in Epidemiology) guidelines [21] and sound ethics principles for conducting and reporting of observational studies [22]. There were a number of study limitations. First, the only surgical outcome assessed was intra-operative mortality. Other useful indicators might have included post-operative infection rates, other post-operative complications and peri-operative mortality. Unfortunately however, these could not be comprehensively assessed due to major contextual constraints in patient follow-up. Second, given the inherent limitations of an observational study like this, we cannot directly attribute the low intra-operative mortality rate to the measures implemented to ensure a minimum standard of care. Finally, data on patient referrals to Turkey were not routinely collected and therefore could not be reported on. Despite these limitations, the study raises a number of issues that merit discussion, particularly in relation to the provision of quality surgical care in conflict settings.

First, although surgical care in conflict settings has often been considered to be primarily indicated for war victims, our study shows that in addition to combat-related cases, a large number of cases are non-combat related, tending to represent the regular burden of surgical disease found in the general population e.g., obstetrical emergencies and infections. This corroborates the findings of other studies from conflict settings [3, 11]. In fact, non-combat related surgical needs are likely to be even greater during times of conflict due to the population being more exposed to factors such as poor hygiene, infectious diseases and poor access to obstetric care [23]. In this regard it is essential that surgical teams who come to operate in such settings come prepared (i.e., have an appropriate team of surgeons and the necessary surgical equipment) to manage both combat and non-combat related surgical cases such as obstetric emergencies.

Second, although we cannot attribute the low intra-operative mortality seen in our study to the surgical intervention itself, our outcomes are extremely favourable when compared to the outcomes reported by another study conducted in a clandestine field hospital in Syria [17]. In this study, among the 28 patients operated on - the most common procedures being vascular procedures, orthopaedic procedures and abdominal exploration - two patients died intra-operatively (giving an intraoperative mortality rate of 7 %). Although we can only speculate, the significantly lower intra-operative mortality rate seen in our study may be due to a number of factors including i) patients dying before arriving at the MSF hospital, ii) better quality of surgical care in the MSF hospital, and/or iii) less severe cases making up the MSF surgical caseload (severe cases being referred to Turkey for management). This latter point raises an important issue - namely the importance of surgical teams recognising when they do not have the capacity to manage particular cases. Patients can pay a high price for sub-standard surgical care - post-operative infections, disability and even death - and thus the principle of ‘do no harm’ should prevail at all times. When it cannot be upheld, we would strongly assert that such surgery should not be undertaken; this is when a good referral system to higher level care becomes a key responsibility of the surgical team.

Third, while recognising the limitations inherent with an observational study like ours, together with the fact that our only outcome measure was restricted to intra-operative mortality, the constraints of this particular context make it very difficult to suggest a more suitable study design and to propose that additional outcome measures should have been assessed. When circumstances allow, we would strongly recommend that indicators such as post-operative infection rates, post-operative complications and peri-operative mortality be measured in order to better assess the overall effect of a surgical intervention. In the context of Syria however this was not possible due to a number of factors, including: i) the need to discharge patients as early as possible-because of both the limited number of hospital beds and the high security risk of keeping patients at the hospital - thus limiting in-patient monitoring time, ii) patients being unable to return or be referred back to the hospital for follow-up due to insecurity and transportation constraints, and iii) patient tracing and follow-up via cell phone not being possible due to the disruption of telephone signal.

The difficulties around post-operative monitoring and patient follow-up are added justification for MSF’s insistence that minimum standards of surgical care be strictly adhered to. According to the concepts outlined in the Donabedian Model, quality of healthcare can be considered in relation to three domains: “structure,” “process,” and “outcomes” [24].” Structure” describes the factors that affect the context in which care is delivered (e.g., hospital buildings, equipment, human resources); “process” describes the transactions between patients and healthcare providers during healthcare delivery (i.e., those factors that influence how healthcare is delivered); and “outcomes” refers to the effects of healthcare on the health status of patients/populations. The minimum standards of surgical care endorsed by MSF ensure that all the necessary “structural” elements are in place for maximising quality of care. Meanwhile, MSF ensures that important ‘process’ factors are upheld through comprehensive and standardised protocols for all levels of care, appropriate record keeping and comprehensive data collection systems. In conflict settings, such as Syria, where outcome measurement - as a reflection of quality of care - is often going to be a challenge, we would advocate that when minimum standards of care are rigorously endorsed, and guidelines and protocols strictly adhered to, we can reasonably assume that patients are receiving good quality care and that the results of our activities are satisfactory. This rationale is supported by the fact that across different MSF surgical projects (where there are notable differences in context, surgical caseload, and surgical capacity, but where the same minimum standards of care are strictly adhered to), intra-operative mortality and post-operative rates are consistently low [25].

Finally, based on MSF’s experience of providing surgical care in Syria, there are a number of lessons learnt and practice implications that could help to inform other actors who set out to provide surgical care in conflict settings like Syria. First, the demands are high: surgical centres often need to be erected in a short time frame and usually from scratch, and temporary structures are often the only feasible option which poses a number of challenges particularly in relation to ensuring a minimum quality of care. Second, pertaining to the principle of ‘do no harm’ we would strongly recommend that surgical activities only be offered if a specific complement of minimum measures can be implemented to assure quality of care (adequate infrastructure, a clean and sufficient water supply, effective sterilisation, comprehensive guidelines and protocols etc.). This is only likely to be possible when innovative, adapted and well-organised ways of working can be implemented, supported by highly experienced staff. Third, beyond the provision of surgical activities, appropriate referral networks should ideally be identified where possible, so that patients who cannot be managed due to resource constraints can be referred for appropriate care. Related to this, complimentary support (e.g., the supply of specific surgical materials) to other actors in the field who have the human resource capacity to provide general and more specialised surgery can improve overall access to a greater scope of surgical care. Finally, a certain level of preparedness is essential, including having i) policies on minimum standards according to best practice pre-established, ii) standardised protocols for all levels of care (safety, infection control, surgical etc., iii) standardised monitoring and evaluation tools in place so that reliable data can be collected [26]- including, where possible, patient follow-up data (infection rates, complications, peri-operative mortality) and data on patient referrals, iv) a pool of surgical personnel to recruit from, and v) the surgical capacity to manage both war-wounded and non-trauma emergency cases, such as obstetrical emergencies.

## Conclusion

In conclusion, surgical needs in a conflict-afflicted setting like Syria are high and include both combat and a large number of non-combat indications, particularly obstetric emergencies. Provision of quality surgical care in a volatile and complex setting like this is possible providing appropriate measures, supported by highly experienced staff, can be implemented that allow a specific set of minimum standards of care to be adhered to. This is particularly important when patient outcomes - as a reflection of quality of care - are difficult to monitor and assess.
